# Resveratrol Prevents EBV Transformation and Inhibits the Outgrowth of EBV-Immortalized Human B Cells

**DOI:** 10.1371/journal.pone.0051306

**Published:** 2012-12-10

**Authors:** J. Luis Espinoza, Akiyoshi Takami, Ly Quoc Trung, Shunichi Kato, Shinji Nakao

**Affiliations:** 1 Cellular Transplantation Biology, Kanazawa University, Kanazawa, Japan; 2 Department of Hematology and Oncology, Kanazawa University Hospital, Kanazawa, Japan; 3 Department of Cell Transplantation, Tokai University School of Medicine, Isehara, Japan; Duke University Medical Center, United States of America

## Abstract

**Background:**

Epstein Barr virus-associated lymphoproliferative disease is an increasing complication in patients with immunosuppressive conditions. Although the current therapies for this disorder are effective, they are also associated with significant toxicity. In an attempt to identify newer therapeutic agents, this study investigated the effects of Resveratrol, a naturally occurring polyphenolic compound, on the EBV transformation of human B cells.

**Methodology/Principal Findings:**

This study demonstrates that resveratrol prevents EBV transformation in human B cells. These effects are mediated by specific cytotoxic activities of resveratrol against EBV-infected B cells that are associated with the downregulation of the anti-apoptotic proteins Mcl-1 and survivin. This occurs as a consequence of the inhibition of EBV-induced NFκB and STAT-3 signaling pathways and a resveratrol-induced decrease in the expression of the oncogenic viral product LMP1 in EBV-infected B cells. In addition, resveratrol decreased the expression of miR-155 and miR-34a in EBV-infected B cells, blocked the expression of the anti-apoptotic viral gene BHRF1, and thus interrupted events that are critical for EBV transformation and the survival of EBV-transformed cells.

**Conclusions/Significance:**

These results suggest that resveratrol may therefore be a potentially effective therapeutic alternative for preventing EBV-associated lymphoproliferative diseases in immune compromised patients.

## Introduction

Post-transplant lymphoproliferative disorder (PTLD) is a life-threatening complication that develops as a consequence of ineffective T-cell function due to immunosuppressive therapy in recipients of hematopoietic stem cell (HSCT) or solid organ transplantation (SOT). PTLD is associated with Epstein-Barr virus (EBV) infection of B cells and encompasses a heterogeneous group of disorders ranging from benign mononucleosis-like illnesses to aggressive non-Hodgkin’s lymphomas [1].

EBV is an oncogenic herpes virus that is linked with several malignant disorders, including Hodgkin’s lymphoma, Burkitt’s lymphoma, gastric cancer and nasopharyngeal carcinoma [2]. The EBV genome is a 172 kb double-stranded DNA linear molecule that encodes more than 100 viral proteins, although in latent infected cells only a limited number of gene products are expressed and these include six EBV nuclear antigens (EBNA1, -2, -3A, -3B, -3C, and -LP), three latent membrane proteins (LMP1, -2A, -and 2B), and two small nonpolyadenylated RNAs EBER 1 and 2 [3]. Two viral products, EBNA2 and LMP1, are absolutely required for the *in vitro* transformation of B cells whereas EBNA1, EBNA3A and EBNAC are not indispensable, but nevertheless play a crucial role in the B cells transformation process [3]. Other EBV factors, including the viral Bcl-2 analogous BALF1 and BHRF1, also play critical roles in B-cell transformation since their very early expression after infection, prevents EBV-infected B cells from undergoing apoptosis [4].

Primary EBV infection, which usually occurs in childhood, is generally asymptomatic; however EBV infection may cause infectious mononucleosis when acquired in adolescence or adulthood. EBV persists for life in the memory B cells compartment and reactivation of EBV is prevented by an effective immunosurveillance mediated through virus-specific T-cell immunity [5]. However, infected B cells can proliferate without control in the absence of an effective immune response, resulting in malignant transformation. This process is exemplified by the development PTLD or lymphoproliferative disorders in patients undergoing immunosuppressive therapy for other medical conditions [6], [7]. Currently, reactivation of EBV is an increasing complication in immune deficient patients, particularly after HSCT or SOT [7]. Whereas the tapering of immunosuppression, donor lymphocyte infusion, and rituximab may be effective for this complication after HSCT, severe adverse events such as fatal graft-versus-host disease and infections could develop thereafter [8], [9], therefore effective but less toxic anti-EBV therapies are needed.

Resveratrol (3, 4′, 5 tri-hydroxystilbene) is a naturally occurring polyphenol found in red wine, grapes and other sources [10]. Numerous health benefits have been associated with resveratrol including anti-inflammatory, anti-aging and antitumor activities [10], [11]. The broad anti-cancer activities of resveratrol are exerted by a particular property of this compound to target multiple proteins including the NFκB, STAT-3, and AKT pathways which are all involved in the regulation of cell proliferation, survival and apoptosis [11]–[13]. Several studies have reported inhibitory activities of resveratrol against various virus including herpes simplex virus 1 and 2, Varicella zoster virus, human cytomegalovirus, and influenza virus, however, the mechanisms for such antiviral properties have not been clearly defined [14], [15].

The current study investigated the potential antiviral effects of resveratrol against EBV, and focused on the modulation of the oncogenic properties of this virus. This study took advantage of the ability of EBV to transform B cells into lymphoblastoid cell lines (LCL) *in vitro*, which recapitulates many aspect of the EBV-related malignant transformation. The data revealed that resveratrol effectively prevented the EBV-induced transformation of B cells, which was mediated by the inhibitory effects of resveratrol on multiple genes involved in transformation and survival of human B cells. This is the first report to show mechanistic insights into the efficacy of resveratrol to prevent EBV-related transformation and the proliferation of human B cells.

**Figure 1:**
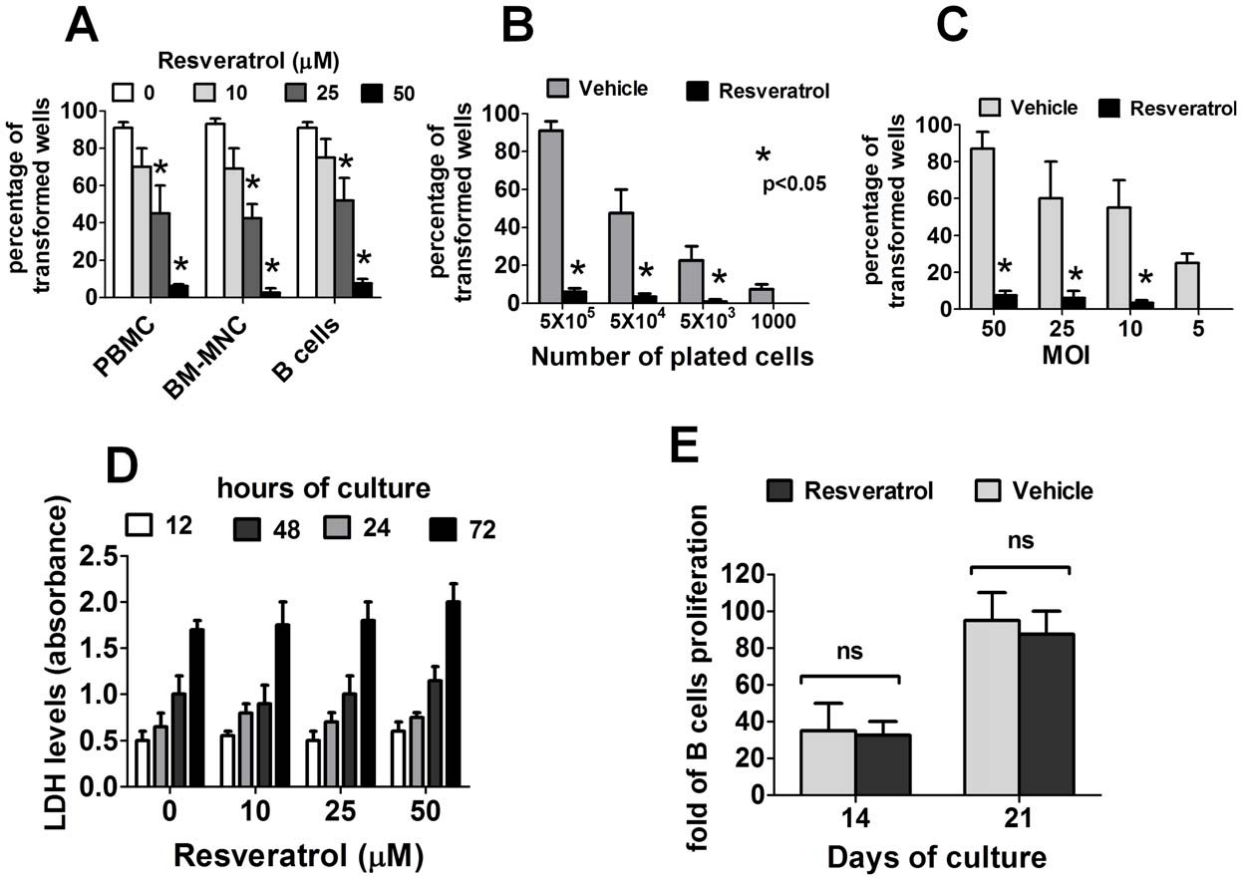
Resveratrol prevents the EBV transformation of human B cells. (A) PBMC (7 donors), CB-MNC (3 donors) or purified B cells (3 donors) were infected with EBV (50 moi) and cultured with vehicle (DMSO 0.05%) or with increasing concentrations of resveratrol. (B) PBMCs (from three donors) were infected with EBV (50 moi) and then seeded at three-fold cell limiting dilutions into replicate wells of a 96-well plate and cultured in the presence or absence of resveratrol (50 µM). (C). PBMCs (from three donors) were infected with a range of virus dilutions and cultured in the presence or absence of resveratrol (50 µM). In A, B and C, the number of wells containing EBV-transformed cell clones was assessed with microscopic inspection six weeks after infection. Bars and error bars represent mean±SEM of experiments performed with cells from the indicated number of donors. (D) Purified B cells were cultured in the presence or absence of several concentrations of resveratrol. The release of LDH in the cell supernatants harvested at the indicated times was measured using a LDH detection kit. (E) PBMCs (5×10^5^/well) were stimulated with IL-4 and recombinant soluble CD40L in the presence or absence of resveratrol (50 µM). After 14 and 21 days of culture, the number of CD19^+^ B cells was assessed using flow cytometry. In figures D and E the mean±SEM of experiments using cells from three different donors is shown.

## Materials and Methods

### Cell Lines

The EBV producing cell line B95-8 was obtained from the American Type Culture Collection (Rockville, MD) and they were cultured in RPMI 160 medium supplemented with 10% FBS and 1% penicillin and streptomycin. The Akata cell line carrying the EGFP-EBV [16], which is a Burkitt lymphoma-derived cell line [17] were obtained from Dr K. Takada (Hokkaido University). These cells were cultured in RPMI 160 medium supplemented with 10% FBS and 500 µg/ml G418 as described [16].

**Figure 2:**
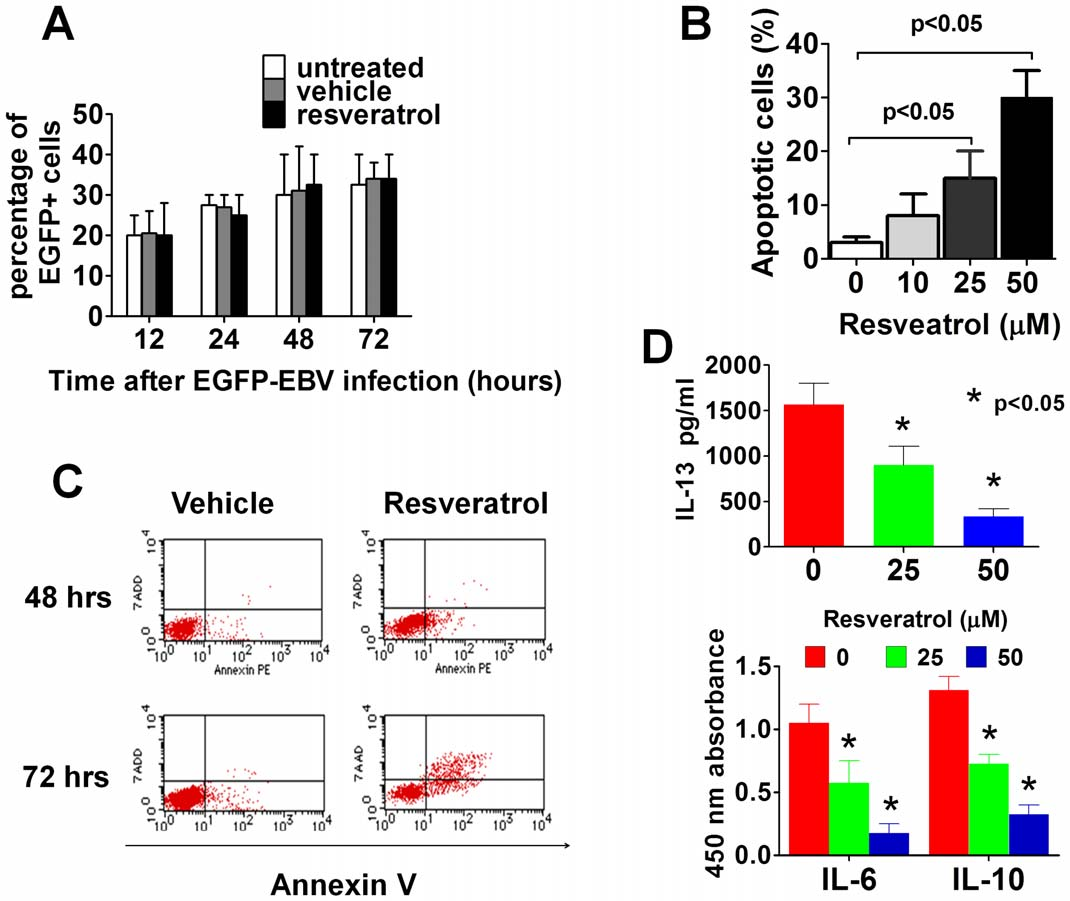
Resveratrol does not block the entry of EBV into B cells but induces apoptosis in EBV infected cells. (A) Primary B cells were infected with EBV-GFP and cultured in the presence or absence of resveratrol (50 µM). The infected cells were collected several times after infection and the percentage of cells expressing EGFP (EBV-infected B cells) was assessed using flow cytometry. Summarized data from three different donors is shown. (B) Purified B cells were infected with EBV-GFP and cultured for up to 96 hours with vehicle or the indicated dose of resveratrol and examined using flow cytometry to quantify the proportion of apoptotic cells. Summarized data using B cells from three different donors is shown and the error bars are the means±SEM of the percentage of apoptotic cells (C) EBV-GFP-infected B cells were treated with 50 µM of resveratrol and cultured for the indicated times and the number of cells in apoptosis was determined using flow cytometry. A representative result of three independent experiments is shown. (D) B cells were infected with EBV and cultured for 72 hours with or without resveratrol and the levels of cytokines released into the culture medium were measured using ELISA or an ELISA multianalyte assay. Mean±SEM of three independent experiments is shown.

**Figure 3:**
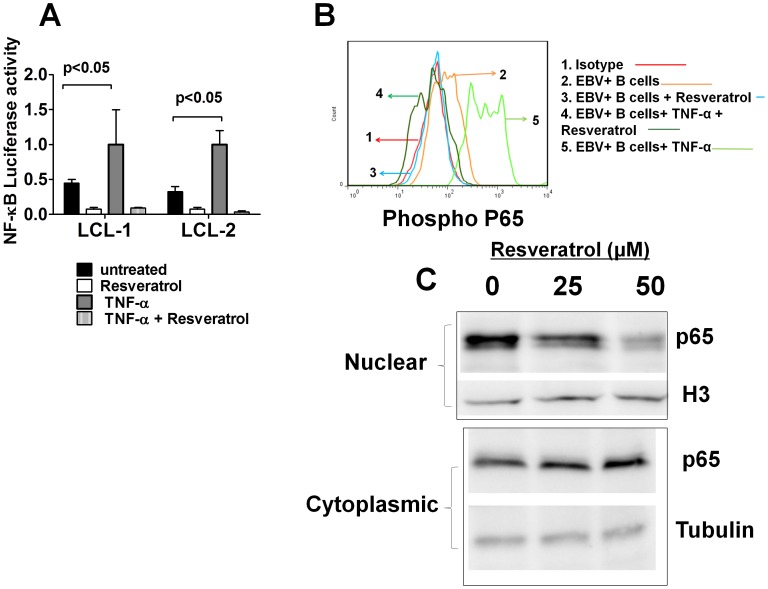
Resveratrol inhibits constitutively-active and TNF-α-induced NFκB activation in EBV-immortalized LCLs. (A) Two LCLs carrying a luciferase reporter vector responsive to NFκB activation were left untreated or were treated with TNF-α for 1 hr and thereafter they were cultured for other 6 hrs in the presence of vehicle or resveratrol. The error bars represent the means±SEM of luciferase activity from three independent experiments. (B) Primary B cells were infected with EBV and cultured for 72 hours. They were then collected and pre-treated with TNF-α for 30 minutes and cultured in the presence or the absence of resveratrol (50 µM) for another 6 hours. The NFκB activity was examined by flow cytometry using an antibody specific for phosphorylated p65. A representative figure of three independent experiments is shown. (C) Cytoplasmic and nuclear extracts from control or resveratrol treated EBV-infected B cells were immunoblotted using anti p65 NFκB antibodies. Samples were reprobed using anti-tubulin and anti-histone H3 (H3) antibodies to control for cytoplasmic and nuclear protein loading. A representative figure of three independent experiments is shown.

**Figure 4:**
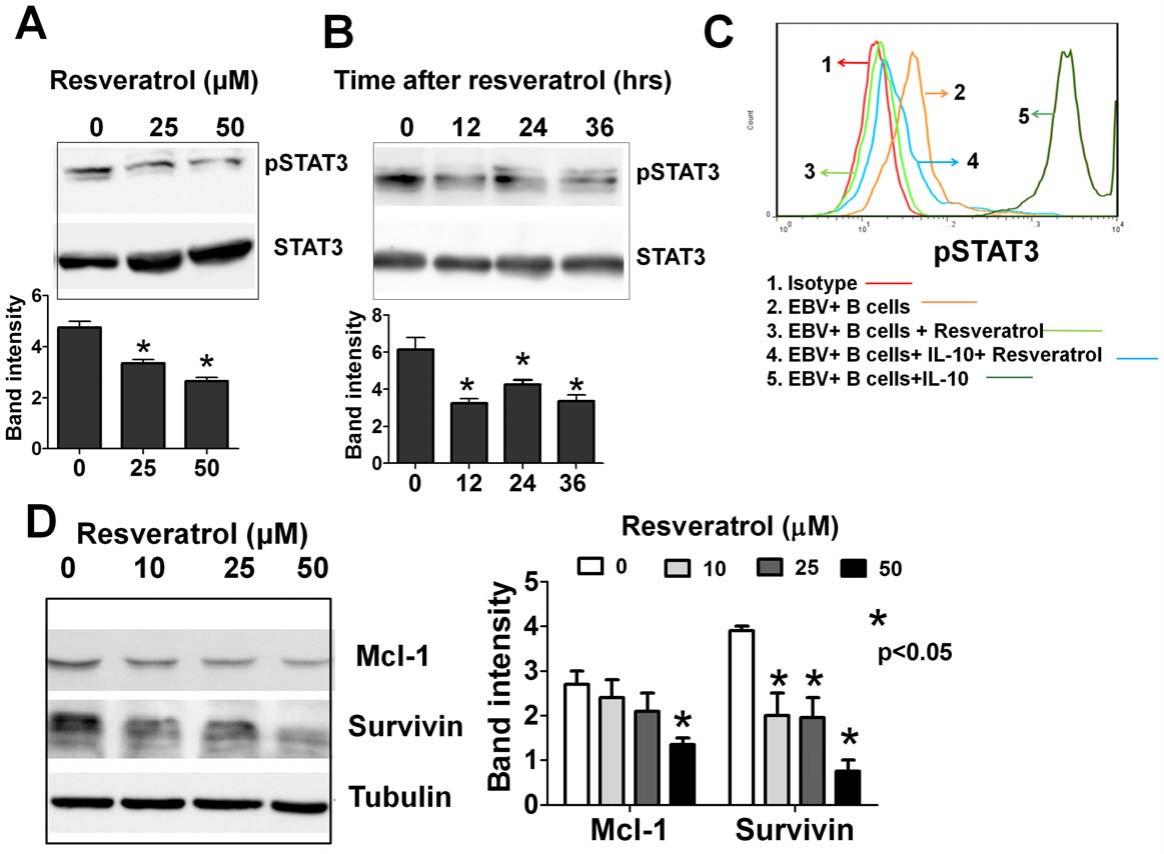
Resveratrol inhibits STAT-3 activation in EBV-infected B cells. (A) Primary B cells were infected with EBV and cultured for 72 hours, then resveratrol was added at the indicated concentrations and the cells were cultured for another 36 hours. (B) Primary B cells were infected with EBV and cultured for 72 hours in the presence of resveratrol (50 µM) and then harvested at the indicated time. In A and B, whole-cell extracts were probed for phospho-STAT-3 using Western blotting. The same blots were stripped and reprobed with anti-STAT-3 antibodies to verify equal protein loading. Representative figures of three independent experiments are shown. Densitometric analysis of the bands obtained in three independent experiments (mean±SEM) is shown in the bottom panel. (C) B cells were infected with EBV and cultured for 72 hours. They were then collected and pre-treated with IL-10 for 30 minutes and cultured in the presence or the absence of resveratrol (50 µM) for another 12 hours, and the expression of phosphorylated STAT-3 was assessed using flow cytometry. Representative figures of three independent experiments are shown. (D) B cells were infected and treated as in panel A and whole-cell extracts were subjected to Western blotting using antibodies specific to Mcl-1 and survivin. The same blots were stripped and reprobed with anti-tubulin antibodies to verify equal protein loading. Densitometric analysis of the bands obtained in three independent experiments (mean±SEM) is shown in the right panel.

**Figure 5:**
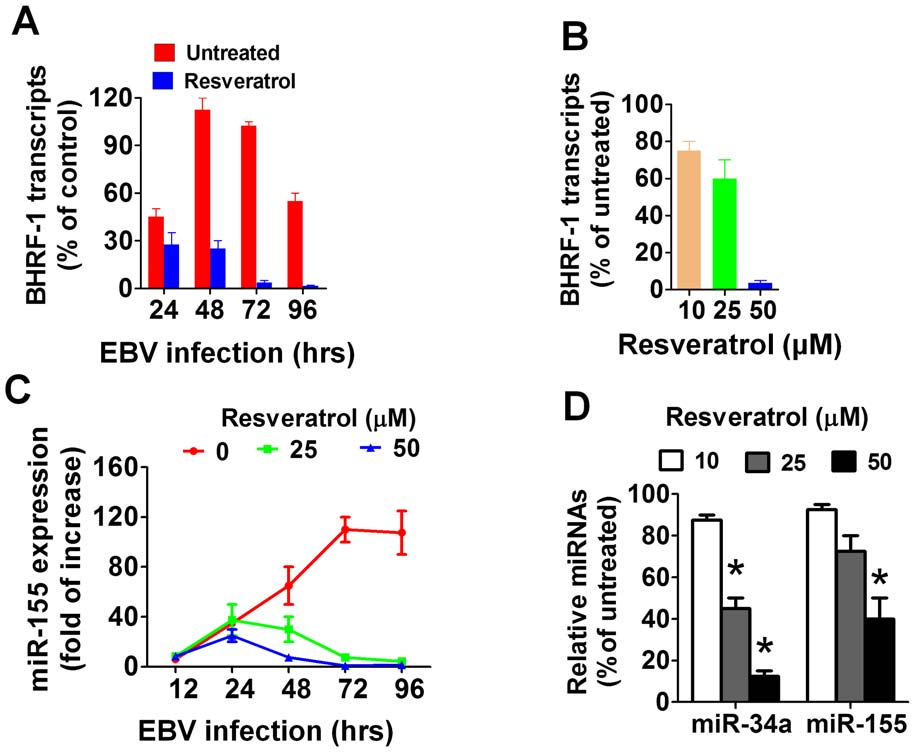
Resveratrol inhibits the expression of viral-induced anti-apoptotic genes in EBV-infected B cells. (A) Primary B cells were infected with EBV and cultured for the indicated times in the absence or presence of resveratrol (50 µM). The level of BHRF1 transcripts normalized to U6B RNA, were measured by qRT-PCR using a lytic infected LCL as a reference control sample. Each data point shown represents the mean±SEM of three independent experiments (B). B cells were infected with EBV and cultured for 72 hours. They were then collected and treated for another 24 hours with or without resveratrol and the BHRF1 transcripts were measured by qRT-PCR (C) EBV infected B cells were cultured for the indicated times in the presence or absence of resveratrol (50 µM) and their levels of miR155 were measured by qRT-PCR (D) EBV-immortalized LCLs (1×10^6^) were treated with resveratrol for 24 hrs and the expression of miR-155 and miR-34a were determined by qRT-PCR. The bars in figures B and D represent the means±SEM of three independent experiments.

**Figure 6:**
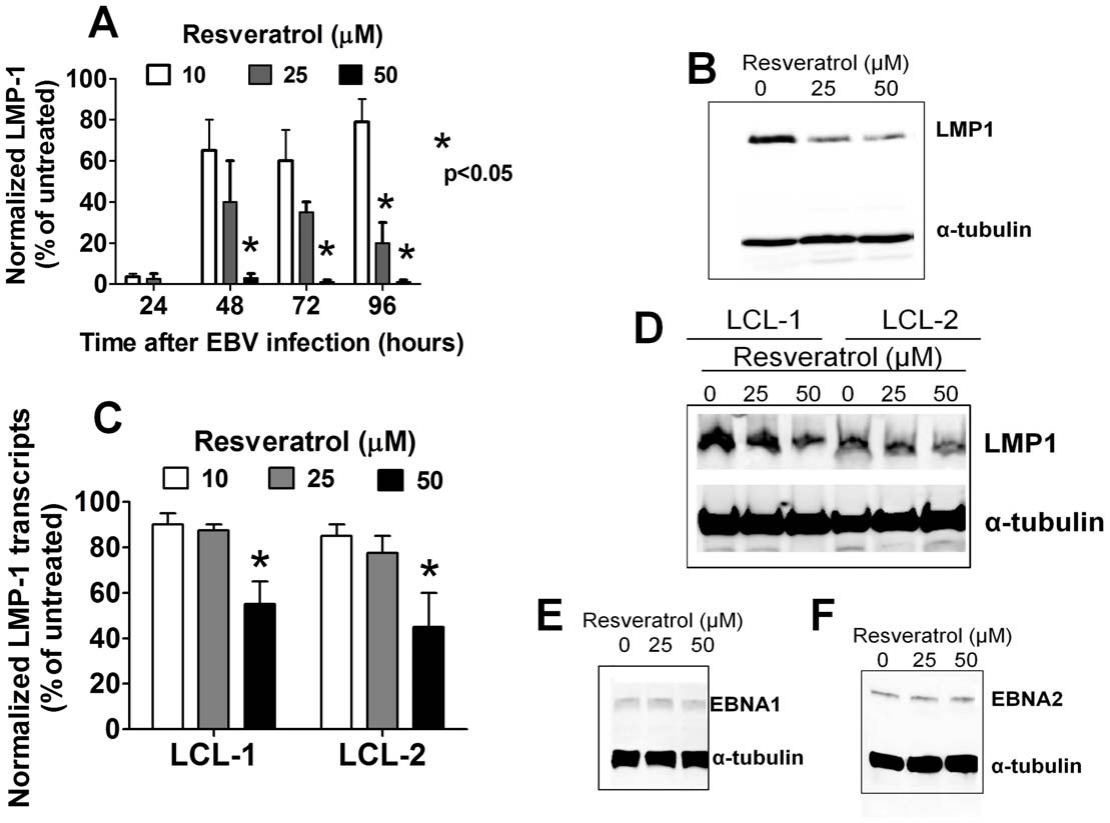
Resveratrol downregulates LMP1 expression in EBV-infected B cells. (A) EBV-infected B cells were cultured for up to 96 hours with the indicated concentrations of resveratrol. Cellular RNA was extracted and the levels of LMP1 transcripts were determined using qRT-PCR. Error bars represent means±SEM of three independent experiments (B) B cells were infected with EBV for 72 hours and then cultured in the presence or absence of resveratrol for another 48 hours, after which the expression of LMP1 proteins was assessed using Western blotting. (C) Two EBV-immortalized LCLs were cultured with or without resveratrol for 48 hours and the levels of LMP1 transcripts were assessed using RT-PCR. Error bars represent means±SEM of three independent experiments (D) Whole-cell proteins from LCLs that were treated as in panel C were subjected to Western blotting and probed with anti-LMP1 antibodies. A representative figure of three independent experiments is shown. LCLs were treated as in panel C and the whole-cell protein extracts were analyzed using Western blotting with antibodies specific for EBNA1 (E) and EBNA-2 (F). The figures shown are representative results of three independent experiments.

**Figure 7:**
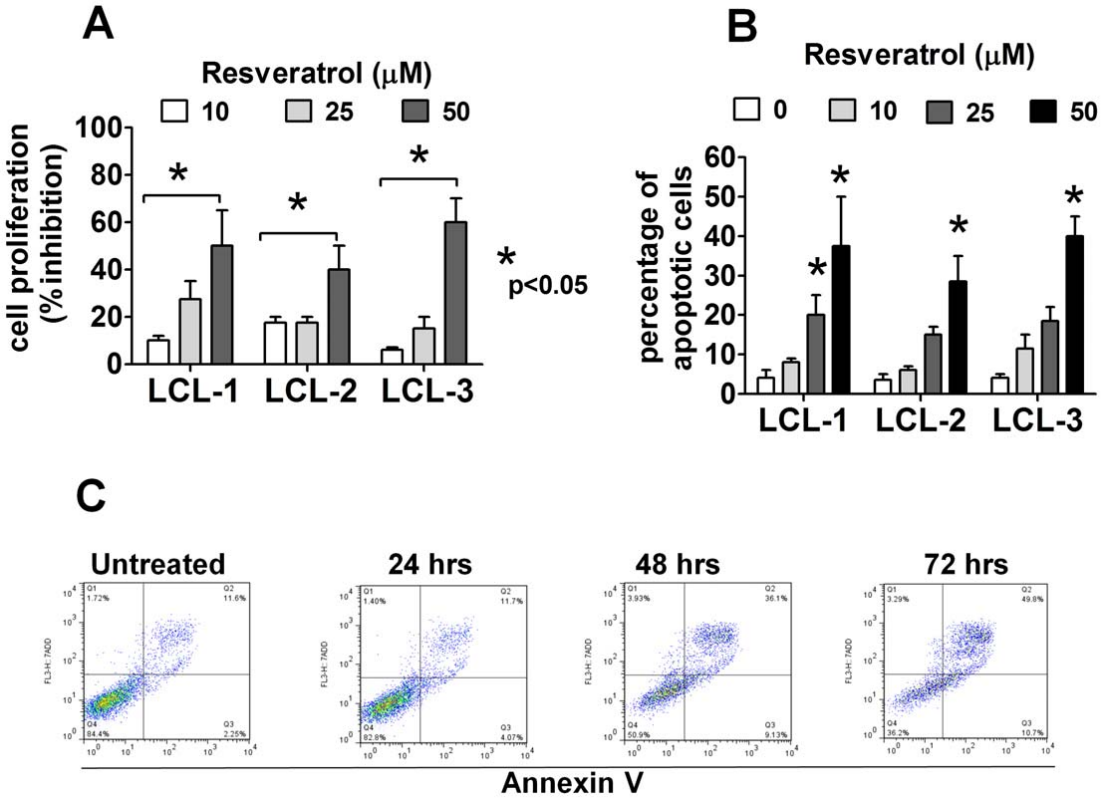
Resveratrol causes cell cycle arrest and induces apoptosis in EBV-immortalized B cells. (A) EBV-immortalized LCLs were cultured for 48 hrs in the presence or absence of resveratrol. The cell proliferation rate was measured by an MTT assay. The figure shows the means±SEM of three independent experiments. (B) EBV-immortalized LCLs were treated with the indicated concentrations of resveratrol and cultured for 48 hrs and the percentage of apoptotic cells were assessed by flow cytometry. Summarized data of means±SEM of apoptotic cells from three independent experiments is shown. (C) EBV-immortalized LCLs were treated with resveratrol (50 µM) and harvested at the indicated time. The percentage of apoptotic cells were assessed by flow cytometry. A representative figure of six independent experiments is shown.

### Reagents

Resveratrol and anti-α tubulin antibody were purchased from Sigma. Hygromycin was obtained from Wako (Tokyo Japan). The NFκB luciferase report vector pGL4.32 [-luc2PNFκB –RE/Hygro] was acquired from Promega. The antibodies directed against survivin, Mcl-1, phosphorylated STAT-3, histone H3 and phosphorylated p65 were purchased from Cell Signaling Technology. Anti-EBNA1 clone 1B5 (Acris Herford, Germany), anti-EBNA2 clone PE2, anti-human IgG and anti-LMP1 clone CS1-4 were from DakoCytomation (Glostrup, Denmark). Recombinant proteins including IL-4, IL-10, soluble CD40 ligand and TNF-α were purchased from PeproTech (Rocky Hill NJ).

### Preparation of EBV Virions

EBV-harboring B95-8 cells at a logarithmic phase were suspended in fresh medium seeded at a density of 10^6^ cells/mL and were cultured for 3 days. EGFP**-**EBV was obtained from the culture medium of Akata cells in which EBV production had been induced by surface immunoglobulin G (sIgG) cross-linking as described previously ( [16]. The culture supernatants were clarified by centrifugation, filtered and stored at −80°C until use.

### Cell Preparation

Blood samples from healthy volunteers were collected under a protocol approved by the Institutional Review Board of the Kanazawa University School of Medical Sciences. Peripheral blood mononuclear cells (PBMCs) derived from the whole blood of 7 healthy volunteers and cord blood mononuclear cells (CB-MNCs) obtained from 3 anonymous donors were isolated by density gradient centrifugation using Lymphoprep (Axis-Shield, Oslo, Norway). Primary B cells were purified from the PBMC fractions of 3 donors by negative selection using magnetic beads *Miltenyi Biotec* (Gladbach, Germany) according to the manufacturer’s specifications. These primary cells were cultured in RPMI 160 medium supplemented with 20% FBS and 1% penicillin and streptomycin designated herein after as culture medium. In experiments using PBMC, the culture medium was supplemented with Cyclosporine A (500 nM/ml; Sigma, Marlborough, MA) to inhibit T-cells immunity.

### EBV-transformation Assays

Three different approaches were used to investigate the effects of resveratrol on B cell EBV transformation efficiency. First, PBMCs, CB-MNC (2×10^6^ cells/mL) or purified B cells (0.5×10^6^ cells/mL) were exposed to a defined virus dose (50 moi) for two hours at 37°C and then seeded in replicate wells of 96-well plates in medium containing several concentrations of resveratrol or vehicle (DMSO 0.05%). In a second set of experiments, cells were infected with a range of virus dilutions before being seeded into a 96-well plate in the presence or absence of resveratrol (50 µM). In another assay, cells were exposed to a defined virus dose (50 moi) and then seeded at three-fold limiting dilutions into replicate wells of a 96-well plate. Fresh medium containing the drug was replaced once per week. In all cases, the percentage of wells containing EBV-transformed cell clones was assessed with microscopic inspection six weeks after infection.

Infection experiments for protein and mRNA analysis were performed by exposing purified B cells (1×10^6^) to EBV and then harvesting the cells at different time points post-infection. LCLs were generated from PBMCs (2 donors) and one CB-MNC donor by infecting 2×10^6^ cells with the supernatant of B95-8EBV and expanded into 25 ml flasks. Cultures were maintained in exponential growth using passaging twice weekly. LCLs were treated with sodium butyrate (5 µM) for 3 days in some experiments, to induce lytic infection and their RNA was extracted and used for quantitative real time PCR.

### Infection of Human B cells with GFP-EBV

Purified B cells (0.5×10^6^) derived from 3 healthy donors were infected with GFP-EBV suspension in 500 µL of RPMI medium supplemented with 10% FBS and incubated for 4 hrs at 37°C. The cells were centrifuged, the supernatant was discarded and then the cells were resuspended in fresh RPMI culture medium in the presence or absence of resveratrol. The infected cells were harvested at several time points and the percentage of GFP expressing cells were determined by flow cytometry. The number of apoptotic cells in the EGFP positive fraction, were assessed by staining the cells with annexin V and 7-AAD followed by flow cytometry analysis.

### B cell Proliferation Assay

Activated B cells were generated as previously described [18] with some modifications. In brief, unseparated PBMCs (5×10^5^/well) from three healthy donors were cultured in RPMI medium supplemented with 20% human serum, 50 ng/ml IL-4 and 10 µg/ml of recombinant soluble CD40L [19] and cyclosporine A in the presence or absence of resveratrol (50 µM). The cells were restimulated every four days with fresh medium containing cytokines with or without resveratrol. After 14 and 21 days of culture, the number of CD19^+^ B cells was assessed using flow cytometry. In some experiments, EBV-immortalized LCL cells (1×10^6^ cells/ml) were plated in 96-well plates and cultured for 72 hours in the presence or absence of resveratrol and assessed for viability and proliferation using an MTT colorimetric assay (Roche, Indianapolis, IN, USA). The inhibition rate of cell proliferation was calculated by the formula: % inhibition = (absorbance of control cells – absorbance of treated cells)/absorbance of control cells×100.

### LDH Assay

Purified B cells (2×10^5^ cells/ml) from three donors were suspended in RPMI 1640 phenol free medium supplemented with 2.5% BSA and 1% FBS, plated in 96-well plates and cultured for 12, 24, 48 and 72 hrs in the presence or absence of several concentrations of resveratrol. The release of LDH in the cells supernatants was measured using the LDH cytotoxicity detection kit (Roche).

### Establishment of EBV-positive Cells Lines Stably Expressing an NFκB Luciferase Vector

The pGL4.32 [-luc2PNFκB –RE/Hygro] vector, designated as NFκB-Luc, which contains five copies of an NFκB response element and drives transcription of the luciferase reporter gene was used to examine NFκB activity in EBV-infected cells lines. The NFκB-Luc vector was transfected into two LCL cells using the GenomONE™-Neo EX HVJ Envelope Transfection Kit (Ishihara Sangyo, Tokyo Japan) according to the manufacturer’s protocol. Stable transfectants were established by selection with hygromycin B (300 µg/ml).

### NFκB -Luciferase Activation Assay

The cell lines stably expressing the NFκB-Luc vector were cultured for 6 hrs in the presence or absence of variable concentrations of resveratrol. Some cells were pretreated with 20 ng/ml of TNF-α to over activate NFκB signal. The cells were harvested and lysed using the passive lysis buffer (Promega). The luciferase activity in the lysates which represents NFκB activity was quantified using a luciferase system (Promega) in a Mabskan LB system (Thermo Scientific).

### Western Blot Analysis

Whole-cell protein samples were prepared by lysing the cells in M-PER® Mammalian Protein Extraction Reagent (Pierce) supplemented with phosphatase and a protease inhibitor cocktail (Sigma). Nuclear and cytoplasmic fractions were isolated using the NE-PER kit (Pierce). Equal amounts of protein extracts were separated by SDS–polyacrylamide gel electrophoresis (SDS–PAGE), and transferred to PVDF membranes (Millipore). Milk-blocked blots were incubated at 4°C overnight with primary antibodies and then incubated with appropriate horseradish peroxidase conjugated secondary antibodies. The proteins of interest were revealed using the WestPico chemiluminescence system (Pierce) and viewed in a luminescent image analyzer Las-400 mini (Fujifilm). Where indicated comparative blots intensities were assessed using the Image J software (NIH, Bethesda, MD).

### Flow Cytometry Analysis

B cells infected with EGFP-EBV were harvested at 12 hrs, 24 hrs, 48 hrs, 72 hrs, and 96 hrs post-infection and analyzed by flow cytometry. Primary B cells infected with EBV and cultured for 72 hours or primary B cells, that had been stimulated with CD40L/IL-4 for 7 days, were collected and treated for 30 minutes with TNF-α (20 ng/ml) to induce hyperactivation of NFKB or with IL-10 to enhance STAT-3 activity. The cells were then cultured for 6 hours in the presence or the absence resveratrol (50 µM) and then were harvested and fixed with 4% paraformaldehyde followed by permeabilization with 90% methanol and staining with a PE labeled anti NFκB or an Alexa Fluor 488 labeled antibody that recognizes STAT-3 phosphorylation at Tyr-705 (clone D3A7) or the corresponding isotype control antibodies (Cell Signaling Technology). Flow cytometry was carried out on a flow cytometry instrument using the Cell-Quest software package (Becton-Dickinson, San Jose, CA). Data analysis was performed using FlowJo software.

### Cytokine Measurement

Purified B cells were infected with EBV and cultured for 48 hours. They were then plated at a density of 5×10^5^ cells/ml and cultured for other 72 hrs in the presence or absence of resveratrol. The levels of the EBV-related cytokines (IL-6 and IL-10) in the culture supernatants were determined using the Multianalyte Human Cytokine Assay kit (SAB-Qiagen) or using a specific ELISA assay for IL-13 (Mabtech, Nacka Strand, Sweden).

### Quantitative Real Time PCR

Total RNA was extracted using Tripure Isolation reagent (Roche) following the manufacturer’s instructions. Complementary DNA (cDNA) synthesis was carried out using the QuantiTect Reverse Transcription kit (Qiagen Inc. Hilden Germany) which includes a DNAse treatment step to remove any traces of contaminant genomic DNA. Amplification of cDNA was monitored using the SYBR Premix Ex Taq RT-PCR kit (Catalog RR041, Takara Bio, Japan) on a StepOne plus instrument (Applied Biosystems). Specific primers for BHRF1 and EBNA-1 [20], and LMP1 [21], as well as a GAPDH primer set (Takara Bio) were used for mRNA quantification in each sample. The amount of each mRNA relative to GAPDH mRNA was calculated by the comparative CT method using the relative expression function included in the StepOne v2.2 software package (Applies Biosystems). The time kinetics levels of BHRF1 transcripts in EBV-infected B cells were normalized to the BHRF1 levels in a lytic infected LCL and expressed as the percentage of the control.

### Measurement of microRNAs

Total RNA was reverse transcribed using a TaqMan microRNA RT kit (Applied Biosystems) and the resultant cDNA was amplified using specific TaqMan microRNA assays for human miR-155 and U6B RNA with the TaqMan Universal PCR master mix II no UNG (Applied Biosystems). The PCR reactions and cycling parameters were set according to the manufacturer’s recommendations. Specific miScript primer assays for human miRNA-34a, miRNA-1245, miRNA-223 and U6B RNA, all from Qiagen, were used to amplify cDNA samples that were prepared from B cells derived total RNA using the miScript RT kit II (Qiagen). The PCR reactions were carried out using the SYBR Premix Ex Taq RT-PCR kit (Takara). The data analysis was performed with the StepOne v2.2 software package (Applied Biosystems). The relative quantities of those micro RNAs were calculated using the comparative C_T_ method after normalization to the U6B RNA levels.

### Statistical Analysis

All data are reported as the means±S.E. Statistical significance was determined using Student’s *t*-test. The statistical significance of multiple comparisons was determined using a one-way analysis of variance. The data were considered to be statistically significant when p≤0.05. All statistical analyses were performed using the GraphPad Prism software package (San Diego).

## Results

### Resveratrol Prevents the EBV-immortalization of Primary Human B Cells

The ability of EBV to *in vitro* transform B cells in the presence of resveratrol was examined. As shown in **Fig. 1A**, whereas EBV-infected B cells were typically transformed in more than 90% of the wells, resveratrol significantly decreased the transformation efficiency of B cells in a dose dependent manner, ranging from less than 30% of transformed wells in cells treated with 25 µM of resveratrol to less than 5% in cells treated with 50 µM of resveratrol. Resveratrol also impaired the transformation efficiency in conditions under limited cell seeding (**Fig. 1B**) or limited virus dose (**Fig. 1C**). Notably, the concentrations of resveratrol that were effective in preventing EBV-immortalization of B cells were not toxic to normal B cells, as demonstrated by similar levels of LDH in the supernatants of uninfected B cells cultured for up to 72 hours in the presence or absence of resveratrol (**Fig. 1D**) and by comparable proliferation rates in resveratrol-treated and untreated B cells cultured in medium containing recombinant CD40 ligands and IL-4 (**Fig. 1E**).

### Resveratrol Induces Apoptosis in EBV-infected B Cells

PBMC or purified B cells were preincubated with resveratrol and then infected with a recombinant EBV containing enhanced green fluorescent protein gene in its genome (EGFP-EBV). The EGFP-EBV-infected B cells were examined by flow cytometry at several time points after infection to monitor the EBV infection process. No significant differences in the percentage of EGFP positive cells in resveratrol treated or untreated cells were observed through the course of 72 hrs of monitoring purified infected B cells **(** **Fig. 2A**), thus suggesting that resveratrol does not prevent the EBV infection of B cells. However a large number of EBV-EGFP infected B cells treated with resveratrol showed a bizarre shape 72 hrs after infection, and unlike the resveratrol nonexposed cells that formed EGFP-LCLs by three weeks after infection, resveratrol-treated B cells failed to immortalize B cells. Next, purified B cells infected with EGFP-EBV were harvested at 48 and 72 hrs after infection and the number of apoptotic cells in the EGFP-positive (EBV-infected) cell fraction were determined by flow cytometry. Resveratrol induced apoptosis in EGFP-EBV-infected B cells in a dose-dependent (**Fig. 2B**) and time-dependent (**Fig. 2C**) manner, thus suggesting that the anti-EBV effect of resveratrol is mediated by inducing apoptosis in the infected B cells. Since cytokines such as IL-6 and IL-10 contribute to EBV-infected cell survival and proliferation indefinitely [22], culture supernatants of EBV-infected B cells treated with or without resveratrol were assessed for the expression of those and other cytokines. Compared with their untreated counterparts, EBV-infected B cells treated with resveratrol secreted lower levels of EBV-related cytokines such as IL-13 (**Fig. 2D** **top panel**), as well as lower levels of IL-6 and IL-10 (**Fig. 2D** **bottom panel),** thus indicating that resveratrol interfered with the secretion of cytokines, which play a critical role in the survival and proliferation of EBV-infected cells.

### Resveratrol Inhibits the EBV-induced Activation of the NFκB Pathway

The potential inhibition of NFκB activity by resveratrol in EBV-infected cells was investigated since activation of the NFκB pathway is critical for the EBV-transformation of human B cells [23] and resveratrol is an effective NFκB inhibitor in several cellular systems [10], [11]. EBV-immortalized LCLs carrying a luciferase reporter vector, whose activity is detectable only in the presence of active NFκB, were established. **Fig. 3A** shows that TNF-α-induced hyperactivation of NFκB in LCL cells was abrogated by the exposure of these cells to resveratrol. Resveratrol was added to bulk B cells 72 hrs post-infection and the activation status of NFκB was determined by flow cytometry using an antibody specific to phosphorylated p65, a functionally active subunit of NFκB. Resveratrol suppressed both the constitutively active NFκB and the TNF-α-induced hyperactivation of NFκB in EBV-infected B cells (**Fig. 3B** **).** In addition, resveratrol induced a dose dependent decrease in the nuclear translocation of p65 NFκB in EBV-infected B cells (**Fig. 3C**). Taken together these results indicate that the prevention of the EBV-immortalization of human B cells by resveratrol is therefore associated with the inhibition of NFκB.

### Resveratrol Inhibits the EBV-induced Activation of the STAT-3 Pathway in B Cells

The effect of resveratrol on the activation state of STAT-3 was investigated in EBV-infected B cells, since STAT-3 signal activation is implicated in the EBV-mediated oncogenesis [24], and previous studies have shown that resveratrol is an effective STAT-3 inhibitor [25], [26]. Whole-cell protein extracts were collected from EBV-infected B cells at 72 hrs after infection and assayed by Western blotting, with antibodies specific to phosphorylated STAT-3 at Tyr 705. Constitutively active STAT-3 was detected in these cells and their exposure to resveratrol resulted in a dose dependent (**Fig. 4A**) and time dependent (**Fig. 4B**) down-regulation of phosphorylated STAT-3. The inhibitory activity of resveratrol on constitutively active STAT-3 signaling as well as on the IL-10 induced STAT-3 hyperactivation was also demonstrated using flow cytometry with an antibody specific to phosphorylated STAT-3 (**Fig. 4C**). Interestingly, although resveratrol suppressed the IL-10 induced STAT-3 hyperactivation in CD40L/IL-4 stimulated B cells, it showed no inhibitory effect on the constitutively active STAT-3 in those cells **(Fig. S1B)**.

Whole-cell protein extracts from EBV-infected B cells were probed with antibodies specific to Mcl-1 and survivin, since the anti-apoptotic proteins Survivin and Mcl-1, which are downstream of STAT-3 signal activation, are implicated in the survival of EBV-infected malignant cells [24], [27] and resveratrol have been reported to inhibit the expression of anti-apoptotic proteins in other cellular systems [25]. Resveratrol considerably inhibited the expression of Survivin and Mcl-1 in EBV-infected B cells (**Fig. 4D**). These results suggested that the inhibition of STAT-3 and its cascade are involved in the anti-EBV activities mediated by resveratrol. Notably, the expressions of Mcl-1 and survivin proteins in CD40L/IL-4 stimulated B cells were not significantly affected by the resveratrol treatment **(Fig. S1C)**.

### Resveratrol Blocks the Expression of Viral Anti-apoptotic Genes in EBV-infected B Cells

The effect of resveratrol on the expression of BHRF1, an EBV-encoded homolog of the anti-apoptotic protein Bcl-2, was investigated, since the early expression of BHRF1 is critical for EBV-transformation of B cells [4]. Quantitative RT-PCR showed that while BHRF1 transcripts were detectable 24 hrs after infection, BHRF1 transcripts were not detectable in resveratrol exposed cells (**Fig. 5A**), thus indicating that blocking the BHRF1 expression may contribute to the pro-apoptotic properties of resveratrol in EBV-infected B cells. Additional experiments were performed in which resveratrol was started 72 hours after EBV infection and then BHRF1 transcripts levels were measured 24 hours after resveratrol treatment, to rule out the possibility that the absence of BHRF1 mRNAs in resveratrol treated cells was a property of cells undergoing apoptosis and not a specific effect of the polyphenol. **Fig. 5B** shows that resveratrol significantly decreased the BHFR1 transcripts, in a dose dependent manner. In addition, recent reports have shown that EBV infection induces the cellular over-expression of miR-155 and this event is critical for the EBV-transformation of B cells [28], [29] and resveratrol decreases the levels of miR-155 in monocytic cells [30]. Therefore, the effect of resveratrol on the expression of miR-155 was investigated in EBV-infected B cells. Quantitative RT-PCR showed that EBV infection significantly augmented miR-155 expression in B cells at levels that were highest by 72 hrs post-infection. Exposure of EBV-infected B cells to resveratrol however, prevented such a viral-induced miR-155 upregulation (**Fig. 5C**). The levels of other lymphocyte-expressed miRNAs such as miR-1245 [31] and miR-223 [32] were not significantly affected by resveratrol treatment. Surprisingly, the levels of miR-34a, which was recently reported to promote the growing of EBV-transformed B cells [33], were significantly reduced in the resveratrol treated infected B cells (**Fig. S2**). In addition, resveratrol significantly decreased the levels of miR-155 and miR-34a in EBV-immortalized LCLs in a dose-dependent fashion (**Fig. 5D**), thus indicating that the inhibition of EBV-induced over-expression of miR-155 and miR-34a by resveratrol was an important event that contributed to preventing the EBV-immortalization of B cells.

### Resveratrol Decreases the Expression of LMP1 and Induced Apoptosis in EBV-transformed B Cells

LMP1 is the most important oncogenic protein of EBV and is absolutely required for B cells immortalization [2]. PBMC infected with EBV were cultured with or without resveratrol and harvested at several time points after infection. The expression of LMP1 transcripts was determined by quantitative RT-PCR. LMP1 transcripts were detectable by 48 hrs after EBV infection and they remained at high levels for up to 5 days post-infection, as reported elsewhere [34]. Remarkably, resveratrol induced a dose dependent reduction of the LMP1 transcripts (**Fig. 6A**) and significantly decreased the expression of LMP1 proteins in EBV-infected B cells (**Fig. 6B**). In addition, resveratrol treatment also resulted in a decrease in the LMP1 expression at both the transcriptional (**Fig. 6C**) and the protein level (**Fig. 6D**) in EBV-immortalized LCLs. Conversely, resveratrol treatment caused no changes in the expression of other relevant EBV products including EBNA1 (**Fig. 6E**) and EBNA2 (**Fig. 6F**), indicating that LMP1 represents an important target mediating the anti-EBV effects of resveratrol.

Consistent with these findings, resveratrol significantly inhibited the proliferation (**Fig. 7A**) and induced a dose-dependent (**Fig. 7B**) and time-dependent apoptosis (**Fig. 7C**) in three EBV-immortalized LCLs. It may be noted, that the number of apoptotic cells were relatively low in cells treated with resveratrol for 24 hours but it increased dramatically after 48 hours of culture. Taken together, these results indicate that resveratrol may have not only preventive, but also therapeutic, properties against EBV-related malignancies.

## Discussion

Resveratrol is a potent chemopreventive agent with *in vivo* and *in vitro* proved efficacy against a broad spectrum of malignant cells [10]. This report presents a comprehensive study of the anti-EBV activities of resveratrol. The data showed that resveratrol effectively interrupted the immortalization process associated with EBV infection in human B cells and consistently attenuated the proliferation of EBV-transformed B cells. Mechanistic studies showed that such an inhibitory effect of resveratrol on the EBV-associated immortalization was mediated by inducing apoptosis in the EBV infected B cells. Resveratrol inhibited the expression of key EBV genes and blocked viral-induced cellular signals that are essential for the transformation, survival and proliferation of EBV-infected B cells thus; resulting in the apoptosis of the EBV-infected B cells.

EBV uses various strategies to manipulate cellular signal of the host that promotes the survival and the indefinitely proliferation of the EBV-infected B cells. EBV expresses several viral genes during the immortalization process with growth-transforming activity. LMP1 is particularly important because it is a classic oncogenic protein [2] and is indispensable for the EBV-transformation of B cells [3]. Remarkably, resveratrol inhibited the expression of LMP1 gene in EBV-infected primary B cells and consistently down-regulated LMP1 at both the protein and transcriptional levels in EBV-immortalized LCLs. This finding are relevant for understanding the anti-EBV mechanism of resveratrol, since LMP1 functions as a homologue of the constitutively active CD40 receptor and activates NFκB, AKT and STAT-3 [35]–[37], which are pathways involved in the regulation of apoptosis cell proliferation. Notably, the activation of these cascades, particularly NFκB and STAT-3, is necessary for the EBV-immortalization of B cells and is required for the proliferation of EBV-related malignancies [35], [37]. Previous studies showed that resveratrol is an effective inhibitor of the NFκB and STAT-3 pathways, which represents an important mechanism mediating the antitumor properties of this polyphenol [10], [11]. Therefore, it is conceivable that the effective inactivation of NFκB and STAT-3 signals by resveratrol in EBV-infected B cells, occurring as a direct effect of the drug on these targets or as an indirect event associated with the downregulation of LMP1 induced by resveratrol, might account for the efficacy of this polyphenol preventing EBV-immortalization of B cells.

LMP1-dependent activation of NFκB in EBV-infected host cells leads to the production of cytokines including IL-6, IL-8 and IL-10 that contributes to the survival and proliferation of the infected B cells [38]. The present study demonstrated that resveratrol suppressed the secretion of IL-6 and IL-10 in EBV-infected B cells and EBV-immortalized LCLs exposed to resveratrol secreted significant less IL-6, IL-10 and TNF-α than the untreated cells. These events, which appear to be a consequence of the NFκB inactivation induced by resveratrol, may further create a unique microenvironment that does not favor the immortalization and proliferation of the EBV-infected B cells.

A recent report demonstrated the essential role of the viral product BHRF1 in the EBV-transformation of B cells [4]. BHRF1 codes for a homologue of the anti-apoptotic protein Bcl-2 which is highly expressed initially after infection. The very early post-infection expression prevents EBV-infected B cells from spontaneous apoptosis and promotes the cellular transformation [4]. The current data revealed that EBV-infected B cells treated with resveratrol failed to express BHRF1 indicating that the blockade of BHRF1 expression is an important mechanism used by resveratrol to promote the apoptosis in infected cells, thus preventing the EBV-immortalization of B cells.

EBV-encoded LMP1 upregulates the expression of miR-155 through activating the NFκB pathway which interacts with the promoter region of the miR-155 primary transcript [28], hence EBV infection of primary B lymphocytes leads to a sustained elevation of miR-155 and this virally-induced cellular micro RNA miR-155 plays key role in immortalization of B cells by EBV and is essential for the survival of newly generated LCLs [29]. A recent study reported that miR-34a, which is also induced by LMP1 via NFκB activation, promotes the growth of EBV transformed B cells [33]. Therefore, the effective blockade of virally induced miR-34a and miR-155 in infected primary B cells and the downregulation of these miRNAs in LCLs may account for the anti-EBV efficacy of resveratrol. Furthermore, resveratrol-induced downregulation of miR-155 has therapeutic implications given the oncogenic potential of miR-155 and its critical role in rapidly growing EBV-related malignancies such as post-transplant lymphoproliferative disorders [29], [39].

EBV has been etiologically linked to a wide spectrum of malignant diseases [3]. The oncogenic potential of EBV is mainly due to the expression of the LMP1 viral oncogene that functionally mimics CD40 signal activation and elicits powerful proliferative stimuli in the target cells [2], [37] Recently, De Leo et al. reported that resveratrol inhibits the proliferation and survival of EBV-infected Burkitt’s lymphoma cells [40]. These effects were found to be mediated through the inhibition of NFκB and were mitigated to some extent by the latency III EBV. Interestingly, the expression of LMP1 in target cells greatly confers resistance to resveratrol-induced cytotoxicity [40]. In line with those observations, we found that LCLs were resistant to resveratrol during 24 hours of culture; however, resveratrol exerted a robust cytotoxicity against those cells after 48 hours of culture, indicating that a relatively long exposure to the drug is required to eliminate LCLs. It is thus conceivable that by downregulating LMP1 expression in LCLs, resveratrol may overcome the resistance of those cells. Accordingly, reducing the expression of LMP1 with resveratrol might enhance anti-tumor activity in the treatment of LMP1^+^ EBV-associated B cell malignancies, including Hodgkin’s lymphoma, immunoblastic lymphoma and nasopharyngeal carcinoma. Indeed, the chemosensitization of tumors by resveratrol has been demonstrated in other malignancies and is currently under clinical investigation [26].

Resveratrol is virtually non-toxic in humans. Phase I clinical trials have shown resveratrol to be safe and well tolerated at a dose of up to 5 g/day and there are currently more than 20 ongoing clinical trials of resveratrol [41]–[43]. The mean maximal plasma concentrations (C_max_) and half-life times (T_1/2_) of resveratrol (and its active metabolites) in healthy volunteers receiving resveratrol at a daily dose of 5 g were 50 µM and 8 hours, respectively [42]. The effects of resveratrol were significant at concentrations of 25 µM or more in the present *in vitro* study, thus resveratrol is considered to be a clinically promising option to prevent EBV–related B-cell proliferation in HSCT or SOT recipients, especially in those with risk factors for the development of PTLD [1], such as EBV-seronegative recipients and/or donors, lung or heart-lung transplant recipients, those receiving antithymocyte globulin (ATG) or those exhibiting cytomegalovirus disease, T-cell depletion or HLA-mismatching. Patients with an impaired T-cell function, such as those with aplastic anemia treated with ATG and those with primary immunodeficiency, may be alternative candidates for this strategy. Moreover, the observation that resveratrol efficiently eliminates EBV-infected cells by inhibiting the anti-apoptosis pathway of EBV suggests that resveratrol, in combination with currently available therapies, could enhance the effective treatment of EBV-related cancers, including Hodgkin’s lymphoma, Burkitt’s lymphoma, diffuse large B cell lymphoma, nasopharyngeal carcinoma and chronic diseases such as chronic EBV infection or multiple sclerosis [44].

## Supporting Information

Figure S1 **(A) Primary B cells were stimulated with CD40 ligand and IL-4 for 7 days they were collected and cultured for 48 hours in the presence or the absence of resveratrol.** Cell apoptosis was assessed using Annexin V and 7ADD staining and Flow cytometry analysis. A representative figure of three independent experiments is shown. (B) B cells stimulated as in panel A, were collected and pre-treated with IL-10 for 30 minutes and cultured in the presence or the absence of resveratrol (50 µM) for another 12 hours, after which the expression of phosphorylated STAT-3 was assessed using flow cytometry. Representative figures of three independent experiments are shown. (C) B cells stimulated as in panel A, were collected and cultured in the presence or the absence of resveratrol (50 µM) for another 48 hours, after which the expression of Mcl-1 and Survivin was assessed using western Blotting. Representative figures of three independent experiments are shown.
(TIF)

Figure S2 **B cells were infected with EBV and cultured for 72 hours, they were then collected and treated for another 24 hours with or without resveratrol (50 µM) and the levels of several micro RNAs including miR-34a, miR-155, miR-223 and miR-1245 were measured by qRT-PCR. The error bars represent means±SEM of three independent experiments.**
(TIF)
