# Supplementary figures and images for: Resveratrol Prevents EBV Transformation and Inhibits the Outgrowth of EBV-Immortalized Human B Cells
Source: PLoS One. 2012 Dec 10;7(12):e51306. doi: 10.1371/journal.pone.0051306 (PMC3519585; doi:10.1371/journal.pone.0051306)

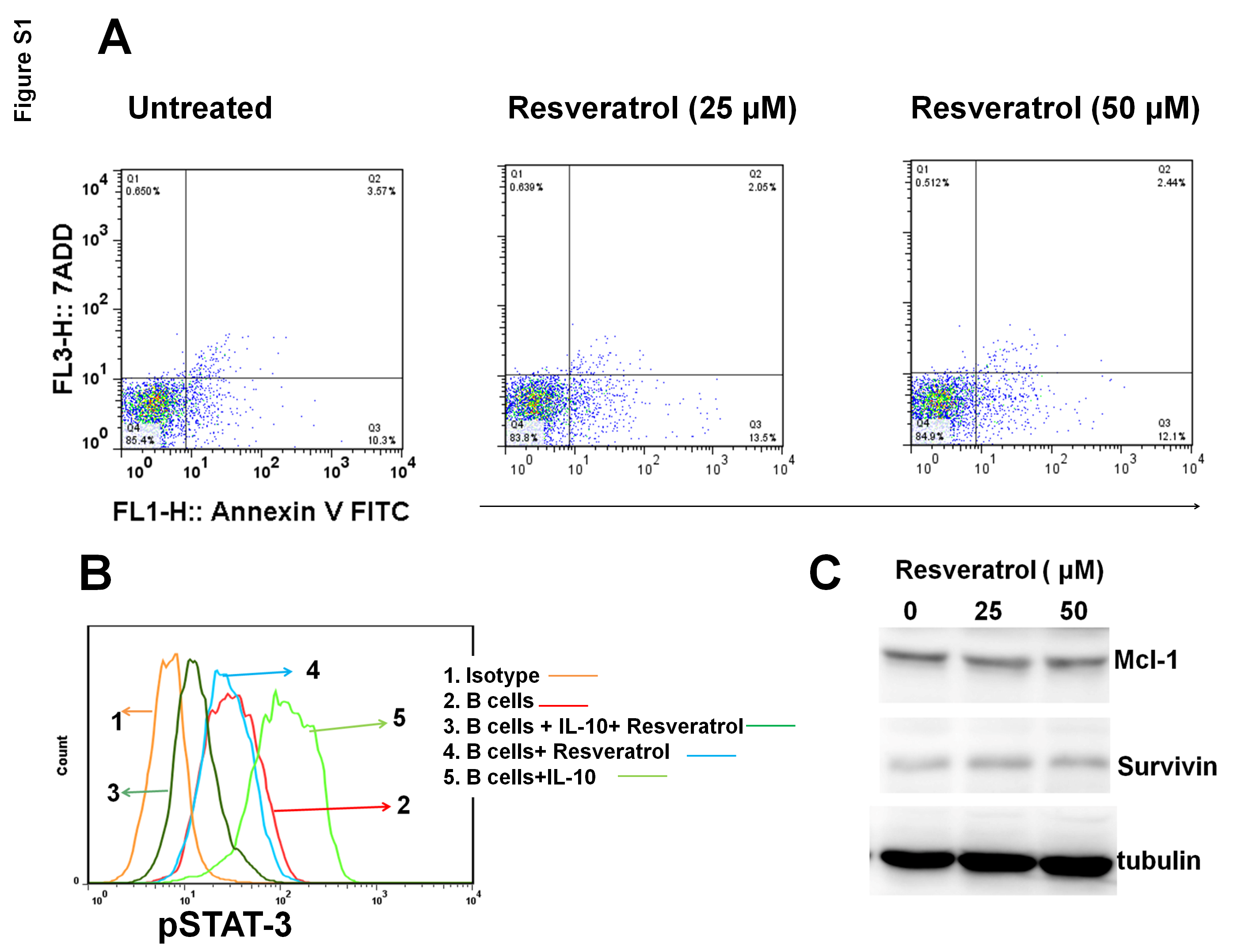

Supplement: Figure S1 — (A) Primary B cells were stimulated with CD40 ligand and IL-4 for 7 days they were collected and cultured for 48 hours in the presence or the absence of resveratrol. Cell apoptosis was assessed using Annexin V and 7ADD staining and Flow cytometry analysis. A representative figure of three independent experiments is shown. (B) B cells stimulated as in panel A, were collected and pre-treated with IL-10 for 30 minutes and cultured in the presence or the absence of resveratrol (50 µM) for another 12 hours, after which the expression of phosphorylated STAT-3 was assessed using flow cytometry. Representative figures of three independent experiments are shown. (C) B cells stimulated as in panel A, were collected and cultured in the presence or the absence of resveratrol (50 µM) for another 48 hours, after which the expression of Mcl-1 and Survivin was assessed using western Blotting. Representative figures of three independent experiments are shown. (TIF) [file pone.0051306.s001.TIF]

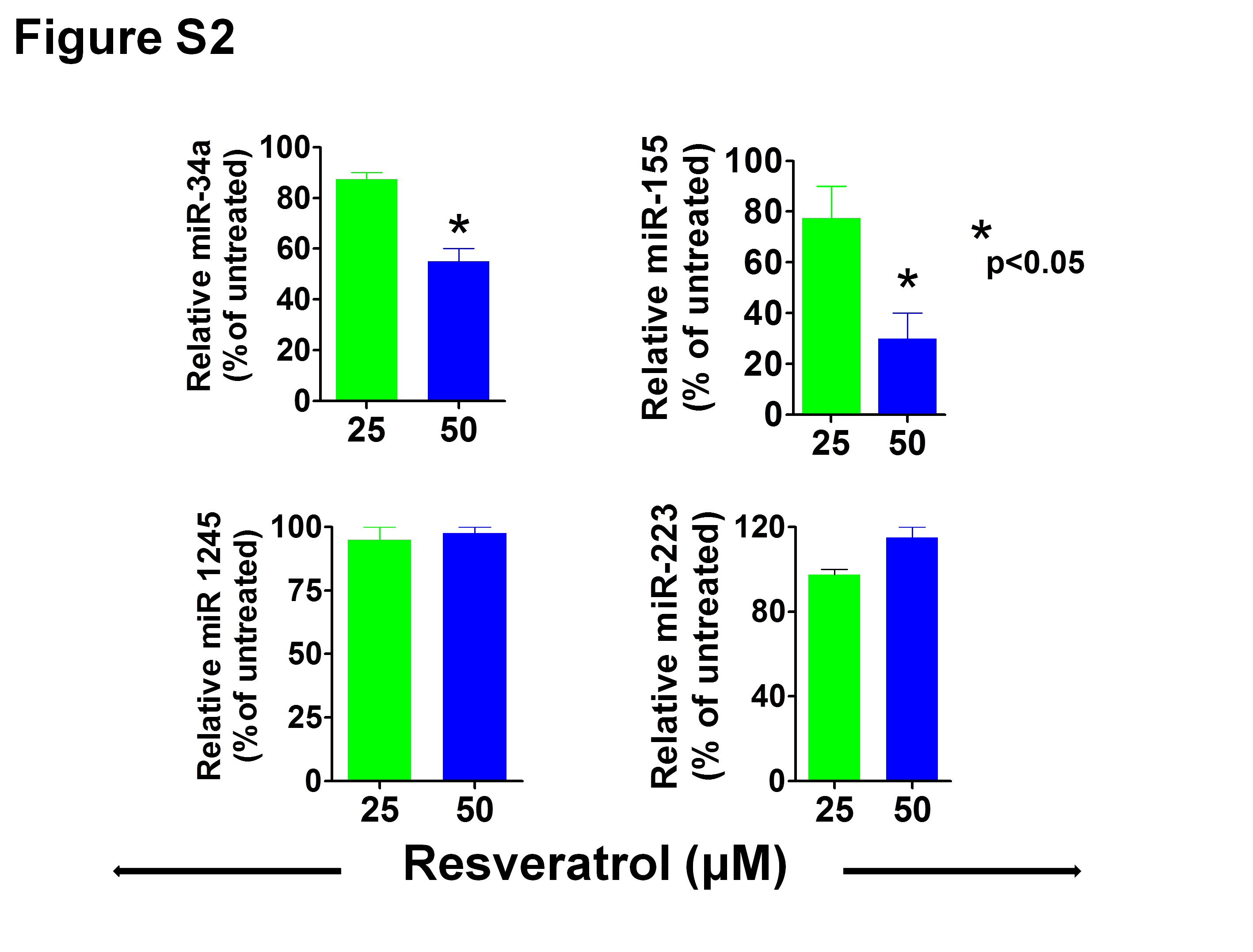

Supplement: Figure S2 — B cells were infected with EBV and cultured for 72 hours, they were then collected and treated for another 24 hours with or without resveratrol (50 µM) and the levels of several micro RNAs including miR-34a, miR-155, miR-223 and miR-1245 were measured by qRT-PCR. The error bars represent means±SEM of three independent experiments. (TIF) [file pone.0051306.s002.TIF]
